# Combined Associations of Serum Ferritin and Body Size Phenotypes With Cardiovascular Risk Profiles: A Chinese Population-Based Study

**DOI:** 10.3389/fpubh.2021.550011

**Published:** 2021-02-15

**Authors:** Bowen Zhou, Siyue Liu, Gang Yuan

**Affiliations:** Department of Endocrinology and Metabolism, Tongji Hospital, Huazhong University of Science and Technology, Wuhan, China

**Keywords:** serum ferritin, iron overload, body mass index, metabolism, cardiovascular disease

## Abstract

**Background:** Serum ferritin (SF) has been correlated with one or more metabolic syndrome features associated with an increased risk for cardiovascular disease (CVD). This study explored the associations between SF and CVD risk factors among different body size phenotypes that were based on metabolic status and body mass index (BMI) categories.

**Methods:** A cross-sectional study was performed using a cohort of 7,549 Chinese adults from the China Health and Nutrition Survey. Participants did not exhibit acute inflammation, were not underweight and were stratified based on their metabolic status and BMI categories. The metabolically at-risk status was defined as having two or more criteria of the Adult Treatment Panel-III metabolic syndrome definition, excluding waist circumference.

**Results:** Compared with individuals without high SF, subjects with high SF had an increased risk of diabetes in the metabolically at-risk normal-weight (MANW) and metabolically at-risk overweight/obesity (MAO) groups. The multivariate-adjusted odds ratios (ORs) were 1.52 [95% confidence interval (Cls): 1.02, 2.28] and 1.63 (95% Cls: 1.27, 2.09), respectively. Adjusted ORs for hyperuricemia from high SF in metabolically healthy normal-weight (MHNW), metabolically healthy overweight/obesity (MHO), MANW, and MAO phenotypes were 1.78 (95% Cls: 1.26, 2.53), 1.42 (95% Cls: 1.03, 1.95), 1.66 (95% Cls: 1.17, 2.36), and 1.42 (95% Cls: 1.17, 1.73), respectively. Similarly, positive correlations of high SF with triglycerides, non-high-density lipoprotein cholesterol, and apolipoprotein B100 were observed in all phenotypes. No association between high SF and elevated low-density lipoprotein cholesterol were observed among participants who were metabolically at-risk, regardless of their BMI categories. However, the ORs for elevated low-density lipoprotein cholesterol from high SF were 1.64 (95% Cls: 1.29, 2.08) in the MHNW group and 1.52 (95% Cls:1.22, 1.91) in the MHO group, significantly. This study demonstrated that the highest ORs were in MAO with a high SF group for all unfavorable CVD risk factors except low-density lipoprotein cholesterol (all *p* < 0.001).

**Conclusions:** The associations of high SF with the prevalence of CVD risk factors, including diabetes, dyslipidemia, and hyperuricemia, vary in individuals among different body size phenotypes. In the MAO group, subjects with high SF levels exhibited worse CVD risk profiles than individuals without high SF.

## Introduction

Serum ferritin (SF) is a storage protein that maintains excess iron in a safe and bioavailable form and correlates linearly with body iron stores (1). Accumulating evidence suggests that there is a correlation between SF levels and one or more metabolic syndrome characteristics (2–6), which are closely linked to insulin resistance (IR) and imply an increased risk for cardiovascular disease (CVD) (7–9). High SF in iron metabolism has been proposed as a component of IR due to its influence on insulin-producing cells as well as insulin-sensitive tissues (2, 10). A prospective cohort study has shown that a significant association between SF and metabolic disturbances remained even after adjusting for homeostatic model assessment of insulin resistance (HOMA-IR), which suggested that alternative underlying mechanisms might exist (11). Another recent study indicated that the SF level was significantly correlated with lipid profiles, independent of hyperglycemia or IR, but the significance of the associations of SF with diabetes and IR were weak or absent after adjusting for dyslipidemia (12). Elevated uric acid (UA) has also been reported to be associated with high SF and metabolic disorders and might be a possible predictor of CVD (13–15). Taken together, the strength of the correlations between SF and cardiometabolic risk factors, such as diabetes, dyslipidemia, and hyperuricemia, might vary due to metabolic status (metabolically at-risk or healthy) and because of disparities in their dependence on IR. Alternative mechanisms, in addition to IR, might be involved. On the other hand, evidence suggests that adipose tissue might play an important role in the process of glucose and lipid metabolism influenced by iron status. The CODAM study observed the association between adipose-tissue IR and iron overload in healthy volunteers (16). An experimental study in mice has reported that an iron-enriched diet led to iron accumulation and IR in visceral adipose tissue (17). Thus, concerning the relationships between SF and CVD risk factors, the different body mass index (BMI) categories (overweight/obesity or normal) and the metabolic status, must be taken into account.

The potential implications of the concomitant presence of metabolic status and BMI categories have triggered interest in comparing these associations with future CVD risk. Individuals were grouped into four different body size phenotypes, including metabolically at-risk overweight/obesity (MAO), metabolically at-risk normal weight (MANW), metabolically healthy overweight/obesity (MHO), and metabolically healthy normal weight (MHNW) (18, 19). Data showed that individuals with MAO experienced the detrimental metabolic profiles characterized by IR, hyperglycemia, hypertension, and atherogenic dyslipidemia, and suffered from an increased risk for CVD (18). Although the associations between the MHO phenotype and cardiometabolic outcomes were not consistent and definition dependent, it should not be considered as a benign status due to its increased risk of metabolic alterations (19).

The correlations between high SF and CVD risk factors mentioned above have not been well-established in different body size phenotypes. The objective of this study was therefore to determine the associations of SF with CVD risk factors according to different body size phenotypes and to assess the combined influence of an unfavorable SF, BMI, and metabolic status on the prevalence of CVD risk factors.

## Materials and Methods

### Study Design and Participants

The China Health and Nutrition Survey (CHNS) is an ongoing prospective cohort survey that includes a total of 10 waves from 1989 to 2015. For each wave, the survey followed a stratified multistage, random cluster process to obtain samples from the provinces and autonomous cities or districts that included approximately half of the Chinese population and significant variation in geography, economic development, and health status. Details about the CHNS are available at http://www.cpc.unc.edu/projects/china/home.html or elsewhere (20). This survey was approved by the institutional review board of the University of North Carolina at Chapel Hill, the National Institute of Nutrition and Health, the China Center for Disease Control and Prevention, and the Human and Clinical Research Ethics Committee of China-Japan Friendship Hospital. Each subject provided written informed consent.

Data relating to 8,704 participants aged 18 years or older were selected from the CHNS 2009, since that was the 1st year blood samples were available. The exclusion criteria included pregnancy (*n* = 62), incomplete information on sex, weight, height, blood pressure (BP), medical history of diabetes and hypertension. We also excluded participants for whom data were missing for one or more of the following values, SF, total cholesterol (TC), triglycerides (TG), high-density lipoprotein cholesterol (HDL-C), low-density lipoprotein cholesterol (LDL-C), apolipoprotein B100 (apoB), apolipoprotein A1 (apoA1), UA, fasting plasma glucose (FPG), and fasting insulin (FIN) (*n* = 251). This study also excluded 349 subjects who exhibited hypersensitive C-reactive protein (hs-CRP) >10 mg/L, which implied the presence of acute inflammation (21). Subjects were categorized as underweight, normal weight, and overweight/obese (with BMI < 18.5, 18.5–22.9, and ≥ 23 kg/m^2^, respectively) in accordance with the World Health Organization criteria for Asian people (22). All underweight individuals (*n* = 493) were removed from the study and a final number of 7,549 participants were included.

### Measurements and Definitions

For each participant, information on age, gender, and medical history of diabetes and hypertension were collected using a structured questionnaire. The participants' weight, height, and resting BP were obtained following strict standard protocols. The BMI was calculated as weight (kg) divided by height squared (m^2^).

Blood samples were collected after an overnight fast. The samples were preserved and analyzed at the national central laboratory in Beijing with standardized protocols. The biochemical parameters including FPG, UA, TC, TG, LDL-C, and HDL-C were measured enzymatically using a Hitachi 7600 automated analyzer (Hitachi Inc., Tokyo, Japan). The level of non-high-density lipoprotein cholesterol (non-HDL-C) was calculated as TC minus HDL-C. Apolipoproteins and hs-CRP were determined using the immunoturbidimetric method (Randox Laboratories Ltd., Crumlin, UK and Denka Seiken Ltd., Niigata, Japan, respectively). SF and FIN concentrations were assessed using radioimmunology (North Institute of Bio-Tech, Beijing, China) *via* an XH-6020 gamma counter. HOMA-IR was calculated as FIN (uIU/mL) × FPG (mmol/L)/22.5.

According to the metabolic syndrome definition of Adult Treatment Panel-III and previous studies (18, 23), individuals were classified as metabolically at-risk when they met two or more of the following criteria: (1) Systolic/diastolic BP ≥ 130/85 mmHg or taking antihypertensive medication. (2) FBG ≥ 5.6 mmol/l or taking antidiabetic medication. (3) HDL-C < 1.0/1.3 mmol/L for men or women, respectively. (4) TG ≥ 1.7 mmol/L. The criterion of waist circumference was not included due to its collinearity with BMI. Participants who met one or no criterion were classified as metabolically healthy. Based on combinations of metabolic status and BMI, subjects were grouped into four different body size phenotypes, MHNW, MHO, MANW, and MAO.

Elevated gender-specific SF levels were defined as SF ≥ the 75th percentile value (≥ 213.20/93.97 ug/L for men or women, respectively), consistent with a definition published in a previous study (24). Individuals were defined as having diabetes if FBG was ≥ 7.0 mmol/L or were previously diagnosed (25). Hypertension was defined as systolic/diastolic BP ≥ 140/90 mmHg or if it was previously diagnosed. Hyperuricemia was defined as having a UA ≥ 6.0/7.0 mg/dL for men or women, respectively (13, 26). Dyslipidemia was defined as TC ≥ 5.2 mmol/L, TG ≥ 1.7 mmol/L, HDL-C < 1.0/1.3 mmol/L for men or women, respectively, LDL-C ≥ 3.4 mmol/L, or non-HDL-C ≥ 4.1 mmol/L, based on the 2016 Chinese Guideline for Adults (27). The presence of unfavorable apolipoproteins was defined as apoB ≥ the 85th percentile value (≥ 1.17/1.19 g/L for men or women, respectively), and apoA1 < the 15th percentile value (0.85/0.90 g/L for men or women, respectively) (28). The inflammation status of participants was categorized as hs-CRP < 1, 1–3, and 4–10 mg/L (29).

### Statistical Analysis

All statistical analyses were performed using SPSS 24.0 (SPSS Inc., Chicago, IL, USA). Data distribution was determined using the Kolmogorov-Smirnov test, and variables were described as medians (interquartile range) or percentages, where appropriate. Based on the cross-classification of the SF status within the four body size phenotypes, the participants were categorized into eight mutually exclusive groups. These specific groups included MAO with or without high SF, MANW with or without high SF, MHO with or without high SF, and MHNW with or without high SF. Differences among the groups were identified using Kruskal-Wallis one-way analysis for continuous variables and Cochran-Mantel-Haenszel chi-square statistics for categorical variables. When it was deemed necessary, a Bonferroni *post-hoc* analysis was used for further multiple comparisons. Logistic regression models were designed to determine the associations of high SF with CVD risk profiles. Multivariable odds ratios (ORs) and 95% confidence intervals (CIs) were adjusted for age, sex, inflammation status (hs-CRP), and HOMA-IR. A bar chart was constructed to delineate the concordance of the three status (metabolically at-risk, overweight/obesity, and high SF) within the whole population and samples with each unfavorable CVD risk factor. Venn diagrams were used to visually display the clustering of the three status in the accumulation of the unfavorable CVD risk factors diabetes, hyperuricemia, and dyslipidemia. Other logistic regression analyses were conducted to evaluate the combined influences of the SF status and body size phenotypes on CVD risk factors. A two-tailed *P* < 0.05 was considered to be statistically significant.

## Results

### Characteristics of Study Subjects Stratified Based on SF Status and Body Size Phenotypes

The prevalence of a high level of SF in the MHNW, MHO, MANW, and MAO phenotypes was 15.9, 21.7, 31.2, and 37.6%, respectively. Sex ratios did not present any significant difference with respect to the SF status within the range of body size phenotypes (*P* = 0.172). Individuals with high SF exhibited higher UA, TG, TC, non-HDL-C, and apoB compared to subjects without high SF in all body size phenotypes (Bonferroni *post-hoc* analysis, *P* < 0.001). When subjects with and without high SF were compared, participants with high SF showed higher FBG for the MHO and MAO groups, higher LDL-C for the MHNW and MHO groups, and lower HDL-C for the MANW group (Bonferroni *post-hoc* analysis *P* < 0.001). Moreover, the differences in FPG, TG, LDL-C, and HDL-C among individuals with and without high SF were significantly influenced by their body size phenotype (*P*-value for the interaction < 0.01). However, the systolic/diastolic BP and apoA1 showed no statistically significant relationship with the SF status for any body size phenotypes (Bonferroni *post-hoc* analysis *P* > 0.05) (Table 1).

**Table 1:** Characteristics of the study participants, stratified by serum ferritin status and body size phenotypes.

	**Metabolically healthy**				**Metabolically at-risk**				***P*^a^**	***P*^b^**
	**BMI < 23 kg/m**^**2**^		**BMI ≥ 23 kg/m**^**2**^		**BMI < 23 kg/m**^**2**^		**BMI ≥ 23 kg/m**^**2**^			
	**Without high SF**	**With high SF**	**Without high SF**	**With high SF**	**Without high SF**	**With high SF**	**Without high SF**	**With high SF**		
*N* (%)	2186 (84.1)	414 (15.9)	1575 (78.3)	436 (21.7)	581 (68.8)	264 (31.2)	1307 (62.4)	786 (37.6)	-	-
Male, %	46.7	48.1	45.3	51.4	45.8	42	48.6	48	0.172	-
Age, years	44.8 (34.9, 56.5)	55.0 (41.7, 64.0)	47.2 (39.6, 56.7)	54.2 (44.0, 62.3)	55.1 (44.6, 66.4)	59.9 (52.9, 67.3)	53.5 (44.6, 62.9)	56.5 (46.7, 63.3)	<0.001	-
BMI, kg/m^2^	21.0 (20.0, 21.9)	21.2 (20.0, 22.1)	25.0 (23.9, 26.6)	25.2 (24.0, 26.6)	21.4 (20.5, 22.2)	21.7 (20.5, 22.3)	25.9 (24.4, 28.0)	26.3 (24.6, 28.2)	<0.001	-
SF, ug/L	53.3 (25.1, 87.7)	238.3 (131.1, 408.7)	57.9 (28.9, 91.5)	248.2 (136.9, 490.7)	66.2 (34.6, 100.3)	232.1 (136.6, 440.7)	70.5 (42.7, 111.1)	268.6 (155.2, 509.5)	<0.001	-
SBP, mmHg	116.0 (108.0, 122.0)	120.0 (110.0, 126.0)	120.0 (110.0, 130.0)	120.0 (114.0, 130.0)	130.0 (120.0, 140.0)	130.0 (120.0, 141.0)	130.0 (120.0, 146.0)	130.0 (120.0, 146.0)	<0.001	0.783
DBP, mmHg	76.0 (70.0, 80.0)	78.0 (70.0, 80.0)	80.0 (74.0, 84.0)	80.0 (74.0, 86.0)	80.0 (76.0, 90.0)	82.0 (78.0, 90.0)	86.0 (80.0, 90.0)	86.0 (80.0, 92.0)	<0.001	0.952
FPG, mmol/L	4.87 (4.56, 5.21)	4.99 (4.62, 5.33)	4.94 (4.63, 5.28)	5.05 (4.69, 5.39)	5.64 (4.99, 6.06)	5.70 (5.04, 6.42)	5.63 (5.07, 6.21)	5.78 (5.25, 6.87)	<0.001	<0.001
UA, mg/dL	4.47 (3.63, 5.45)	4.97 (4.10, 5.85)	4.67 (3.78, 5.63)	5.13 (4.37, 6.23)	4.99 (4.08, 6.20)	5.60 (4.63, 6.71)	5.55 (4.48, 6.70)	6.03 (4.97, 7.24)	<0.001	0.199
TG, mmol/L	0.91 (0.68, 1.23)	1.10 (0.84, 1,41)	1.09 (0.80, 1.44)	1.26 (0.99, 1.57)	1.85 (1.20, 2.59)	2.14 (1.61, 3.20)	2.10 (1.49, 2.92)	2.47 (1.83, 3.78)	<0.001	<0.001
TC, mmol/L	4.49 (3.93, 5.08)	4.85 (4.22, 5.52)	4.73 (4.17, 5.35)	5.16 (4.53, 5.78)	4.80 (4.24, 5.55)	5.09 (4.41, 5.90)	4.98 (4.36, 5.61)	5.31 (4.59, 6.04)	<0.001	0.401
LDL-C, mmol/L	2.71 (2.20, 3.24)	3.01 (2.39, 3.97)	3.00 (2.49, 3.57)	3.29 (2.66, 3.98)	2.88 (2.27, 3.58)	2.95 (2.18, 3.75)	3.04 (2.43, 3.63)	3.08 (2.33, 3.83)	<0.001	<0.001
HDL-C, mmol/L	1.52 (1.32, 1.77)	1.51 (1.33, 1.76)	1.43 (1.24, 1.64)	1.44 (1.26, 1.65)	1.27 (1.09, 1.55)	1.20 (1.04, 1.43)	1.17 (1.00, 1.37)	1.14 (0.97, 1.35)	<0.001	0.006
Non-HDL-C, mmol/L	2.91 (2.40, 3.46)	3.25 (2.69, 3.97)	3.28 (2.74, 3.84)	3.64 (3.07, 4.27)	3.49 (2.88, 4.16)	3.85 (3.14, 4.57)	3.77 (3.18, 4.36)	4.11 (3.46, 4.79)	<0.001	0.790
ApoB, g/L	0.77 (0.64, 0.92)	0.86 (0.73, 1.05)	0.87 (0.73, 1.04)	0.96 (0.80, 1.14)	0.91 (0.76, 1.11)	0.99 (0.79, 1.21)	0.99 (0.82, 1.16)	1.03 (0.86, 1.24)	<0.001	0.065
ApoA1, g/L	1.14 (1.0, 1.33)	1.17 (1.01, 1.34)	1.10 (0.97, 1.27)	1.13 (0.96, 1.29)	1.08 (0.94, 1.27)	1.08 (0.93, 1.30)	1.02 (0.88, 1.21)	1.01 (0.87, 1.21)	<0.001	0.447
Hs-CRP, mg/L	1.0 (0.0, 1.0)	1.0 (0.0, 2.0)	1.0 (1.0, 2.0)	1.0 (1.0, 3.0)	1.0 (0.0, 2.0)	2.0 (1.0, 3.0)	2.0 (1.0, 3.0)	2.0 (1.0, 4.0)	<0.001	0.007
HOMA-IR	1.87 (1.33, 2.61)	1.88 (1.36, 2.64)	2.22 (1.59, 3.20)	2.57 (1.84, 3.61)	2.74 (1.84, 4.09)	2.78 (1.84, 4.98)	3.32 (2.29, 5.42)	3.90 (2.54, 6.72)	<0.001	0.05

*Data are median (interquartile range)*.

a *P represents the difference across SF status within body size phenotypes*,

b *P for interaction terms (SF status × body size phenotypes) were assessed by generalized linear models*.

*BMI, body mass index; SF, serum ferritin; SBP, systolic blood pressure; DBP, diastolic blood pressure; FPG, fasting plasma glucose; UA, uric acid; TG, triglycerides; TC, total cholesterol; LDL-C, low-density lipoprotein cholesterol; HDL-C, high-density lipoprotein cholesterol; Non-HDL-C, non-high-density lipoprotein cholesterol; ApoB, apolipoprotein B100; ApoA1, apolipoprotein A1; Hs-CRP, high-sensitivity C-reactive protein; HOMA-IR, homoeostasis model assessment of insulin resistance*.

### Clustering of Unfavorable SF, BMI, and Metabolic Status

**Figure 1:**
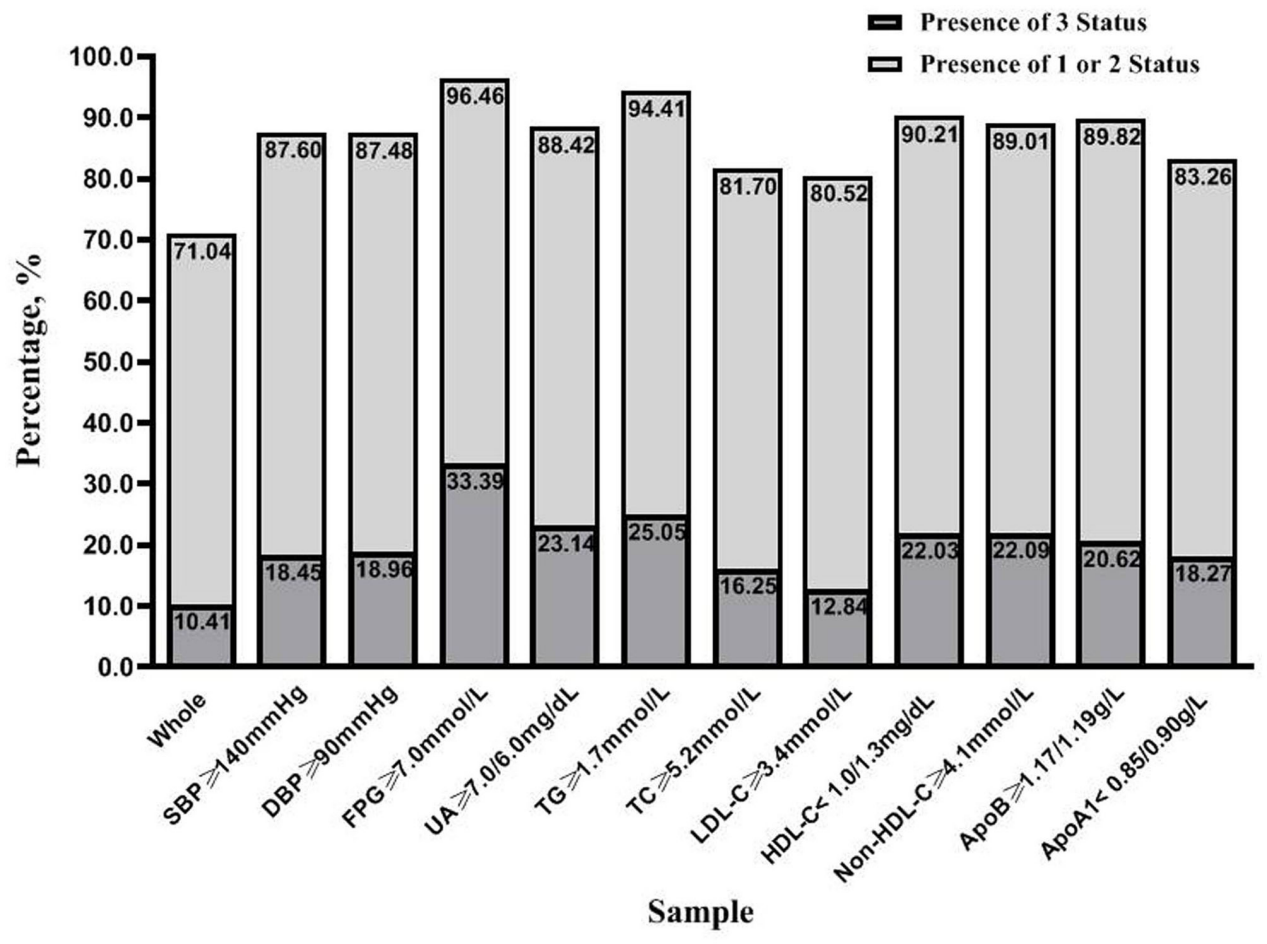
The clustering of the three status (metabolically at-risk, overweight/obesity, and high serum ferritin) in the entire study population and samples with unfavorable risk factors for cardiovascular disease. Each sample was grouped based on the presence of three status and the presence of one or two status. SBP, systolic blood pressure; DBP, diastolic blood pressure; FPG, fasting plasma glucose; UA, uric acid; TG, triglycerides; TC, total cholesterol; LDL-C, low-density lipoprotein cholesterol; HDL-C, high-density lipoprotein cholesterol; Non-HDL-C, non-high-density lipoprotein cholesterol; ApoB, apolipoprotein B100; ApoA1, apolipoprotein A1.

Figure 1 demonstrates the degree of coincidence for the metabolically at-risk status overweight/obesity, and high SF. Of the 7,549 participants, 71.04% were identified as having any of the three status, and 10.41% exhibited all three simultaneously. Similarly, this study found that the three status occurred together in 33.39% of the diabetic participants. For individuals with other unfavorable CVD risk factors, the proportion of participants that exhibited the simultaneous presence of all three status ranged from 12.84 to 25.05%, which was also higher than observed for the total population.

**Figure 2:**
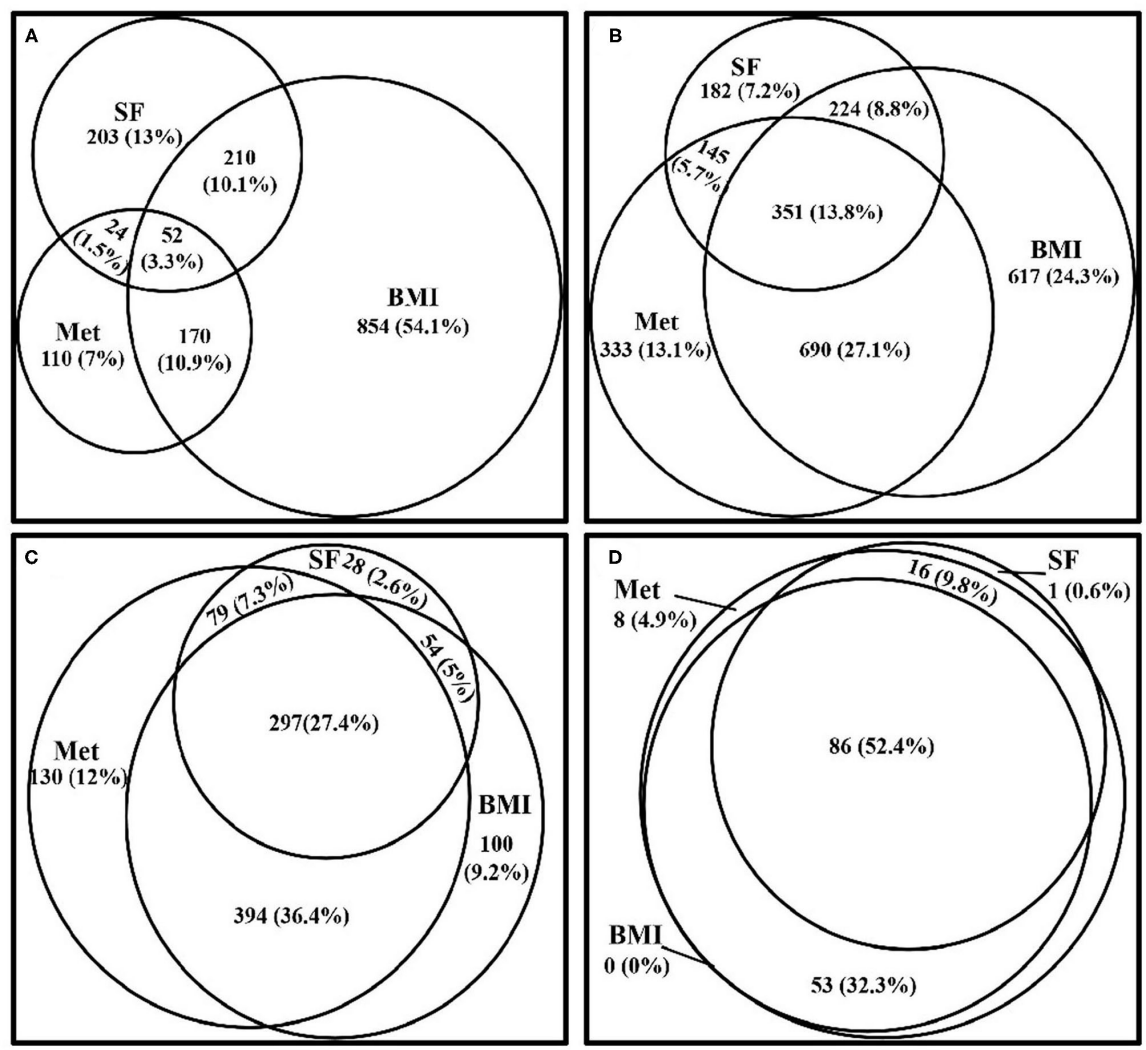
Venn diagrams visually display how the three status (metabolically at-risk, overweight/obesity, and high serum ferritin) cluster together in the samples within 0–3 of the CVD risk factors, diabetes, hyperuricemia, and dyslipidemia, from the CVD risk profiles. **(A)** Venn diagram showing the clustering of the three status in subjects who did not exhibit any of the CVD risk factors. **(B)** Venn diagram showing the clustering of the three status in subjects who exhibited one of the CVD risk factors. **(C)** Venn diagram showing the clustering of the three status in subjects who exhibited two of the CVD risk factors. **(D)** Venn diagram showing the clustering of the three status in subjects who exhibited all three of the CVD risk factors. Met, metabolically at-risk; BMI, overweight/obesity; SF, high serum ferritin; CVD, cardiovascular disease.

Figure 2 depicts the degree of clustering for the metabolically at-risk, overweight/obesity, and high SF status in the samples with 0–3 items of the following CVD risk factors, diabetes, hyperuricemia, and dyslipidemia. The degree of the simultaneous presence of these three status in subjects with 0, 1, 2, and 3 of the CVD risk factors was 3.3, 13.8, 27.4, and 52.4%, respectively. The overlap between the status increased with the presence of increased numbers of CVD risk factors.

### Relationship Between SF and CVD Risk Profiles Among the Body Size Phenotypes

The comparative ORs for high SF and CVD risk profiles for each body size phenotype are shown in Table 2. The association of high SF with diabetes was significant for the MANW and MAO phenotypes. The multivariable-adjusted ORs were 1.52 (95% CI: 1.02, 2.28) and 1.63 (95% CI: 1.27, 2.09), respectively. However, no significance was observed for the association of high SF and diabetes for the MHNW and MHO groups (*P* > 0.05). Compared with individuals without high SF, subjects with high SF exhibited an increased prevalence of hyperuricemia and dyslipidemia (regarding TC, TG, non-HDL-C, and apoB) for all body phenotypes. The adjusted ORs for hyperuricemia in the MAO, MANW, MHO, and MHNW groups were 1.42 (95% CI: 1.17, 1.73), 1.66 (95% CI: 1.17, 2.36), 1.42 (95% CI: 1.03, 1.95), and 1.78 (95% CI: 1.26, 2.53), respectively. Similar results were obtained for dyslipidemia for all body size phenotypes (all *P* < 0.05) except TC (no significance was observed for high SF after adjusting for age, sex, hs-CRP, and HOMA-IR in the MANW group) (*p* = 0.076). There was a more prominent association of high SF with the prevalence of unfavorable non-HDL-C for the metabolically healthy status compared to metabolically at-risk individuals with normal weights (*P*-value for the interaction < 0.05). A high SF was associated with an elevated LDL-C only in the MHNW group (OR: 1.64; 95% CI: 1.29, 2.08) and MHO group (OR: 1.52; 95% CI: 1.22, 1.91). Subjects in this study with a high SF did not exhibit significant ORs for unfavorable systolic/diastolic BP, HDL-C, or apoA1 for any of the phenotypes compared to participants without high SF (all *P* > 0.05).

**Table 2:** Odds ratios (95% confidence intervals) for serum ferritin-related cardiovascular disease risk factors for each body size phenotype.

	**Metabolically healthy**				**Metabolically at-risk**				***P*** **for interaction**	
	**BMI < 23 kg/m**^**2**^		**BMI ≥ 23 kg/m**^**2**^		**BMI < 23 kg/m**^**2**^		**BMI ≥ 23 kg/m**^**2**^			
	**OR^a^**	**OR^b^**	**OR^a^**	**OR^b^**	**OR^a^**	**OR^b^**	**OR^a^**	**OR^b^**	**P_**SF×BMI**_**	**P_**SF×MET**_**
SBP ≥ 140 mmHg	0.96 (0.67, 1.37)	0.60 (0.40, 0.89)	1.32 (1.01, 1.74)	0.97 (0.72, 1.31)	1.26 (0.93, 1.72)	1.04 (0.75, 1.46)	0.96 (0.80, 1.15)	0.83 (0.68, 1.00)	0.076	0.063
DBP ≥ 90 mmHg	1.10 (0.79, 1.54)	0.91 (0.64, 1.28)	1.06 (0.81, 1.40)	0.876 (0.66, 1.16)	1.13 (0.83, 1.55)	1.01 (0.73, 1.39)	0.96 (0.80, 1.15)	0.99 (0.83, 1.19)	0.919	0.699
FPG ≥ 7.0 mmol/L	1.52 (0.61, 3.78)	1.41 (0.55, 3.64)	1.36 (0.63, 2.95)	0.92 (0.40, 2.10)	1.66 (1.13, 2.43)	1.52 (1.02, 2.28)	1.83 (1.47, 2.28)	1.63 (1.27, 2.09)	0.581	0.884
UA ≥ 7.0/6.0 mg/dL (men/women)	1.89 (1.34, 2.67)	1.78 (1.26, 2.53)	1.60 (1.17, 2.17)	1.42 (1.03, 1.95)	1.76 (1.25, 2.49)	1.66 (1.17, 2.36)	1.50 (1.24, 1.81)	1.42 (1.17, 1.73)	0.328	0.825
TG ≥ 1.7 mmol/L	2.02 (1.44, 2.84)	2.12 (1.50, 3.00)	1.58 (1.18, 2.12)	1.57 (1.16, 2.13)	1.65 (1.21, 2.26)	1.87 (1.35, 2.61)	1.84 (1.49, 2.28)	1.89 (1.52, 2.36)	0.155	0.368
TC ≥ 5.2 mmol/L	1.99 (1.59, 2.49)	1.61 (1.29, 2.03)	2.27 (1.83, 2.81)	2.00 (1.60, 2.50)	1.54 (1.15, 2.07)	1.32 (0.97, 1.80)	1.73 (1.45, 2.07)	1.65 (1.37, 1.97)	0.252	0.270
LDL-C ≥ 3.4 mmol/L	2.01 (1.60, 2.53)	1.64 (1.29, 2.08)	1.73 (1.39, 2.15)	1.52 (1.22, 1.91)	1.17 (0.86, 1.60)	0.98 (0.71, 1.35)	1.18 (0.98, 1.42)	1.09 (0.90, 1.32)	0.566	0.012
HDL-C < 1.0/1.3 mg/dL (men/women)	0.70 (0.46, 1.06)	1.02 (0.65, 1.59)	0.57 (0.38, 0.85)	0.96 (0.62, 1.49)	1.14 (0.85, 1.53)	1.25 (0.90, 1.74)	1.11 (0.93, 1.32)	1.15 (0.95, 1.39)	0.429	0.482
Non-HDL-C ≥ 4.1 mmol/L	2.78 (2.11, 3.65)	2.30 (1.73, 3.05)	2.14 (1.68, 2.73)	1.84 (1.43, 2.36)	1.68 (1.24, 2.29)	1.49 (1.08, 2.05)	1.92 (1.60, 2.30)	1.81 (1.51, 2.17)	0.197	0.025
ApoB ≥ 1.17/1.19 g/L (men/women)	2.17 (1.52, 3.09)	1.80 (1.25, 2.59)	2.04 (1.55, 2.67)	1.74 (1.32, 2.30)	1.88 (1.35, 2.63)	1.65 (1.17, 2.33)	1.53 (1.25, 1.86)	1.43 (1.17, 1.75)	0.843	0.674
ApoA1 < 0.85/0.90 g/L (men/women)	0.83 (0.55, 1.25)	0.96 (0.63, 1.47)	0.91 (0.65, 1.29)	1.02 (0.72, 1.46)	1.07 (0.72, 1.60)	1.15 (0.76, 1.75)	1.09 (0.88, 1.34)	1.14 (0.92, 1.41)	0.908	0.538

a *Model was unadjusted*,

b *Model was adjusted for age, sex, inflammation status (hs-CRP) and insulin resistance (HOMA-IR)*.

*P for interaction terms were assessed by logistic regression analyses*.

*BMI, body mass index; OR, odd ratio; SF, serum ferritin; Met, metablic status; SBP, systolic blood pressure; DBP, diastolic blood pressure; FPG, fasting plasma glucose; UA, uric acid; TG, triglycerides; TC, total cholesterol; LDL-C, low-density lipoprotein cholesterol; HDL-C, high-density lipoprotein cholesterol; Non-HDL-C, non-high-density lipoprotein cholesterol; ApoB, apolipoprotein B100; ApoA1, apolipoprotein A1; hs-CRP, high-sensitivity C-reactive protein; HOMA-IR, homoeostasis model assessment of insulin resistance*.

### Joint Effects of SF Levels and Body Size Phenotypes on CVD Risk Profiles

The multivariable-adjusted ORs for CVD risk factors from high SF, overweight/obesity, metabolically at-risk individuals, and combined effects are presented in Table 3. FPG, UA, TG, TC, LDL-C, non-HDL-C, and apoB were chosen for further study based on their significant association with high SF for at least one body size phenotype. Relative to the MHNW without a high SF level, overweight/obesity, and high SF subjects exhibited increased ORs for diabetes when the subjects also were metabolically at-risk. However, if the subjects were not metabolically at-risk, no association was observed between being overweight/obese and having a high SF with a prevalence of diabetes. The status of being metabolically at-risk, overweight/obesity, and high SF increased the ORs for hyperuricemia and dyslipidemia (including TG, TC, LDL-C, non-HDL-C, and apoB). The highest ORs were observed in the MAO with a high SF group for all unfavorable CVD risk factors except LDL-C, and the increase in ORs was greater among participants with high SF.

**Table 3:** Odds ratios (95% confidence intervals)^a^ for joint associations of the SF status and body size phenotypes with the cardiovascular disease risk factors.

	**MHNW**	**MHO**	**MANW**	**MAO**
**FPG ≥ 7.0 mmol/L**				
Without high SF	1	1.55 (0.84, 2.86)	9.97 (5.93, 16.77)	11.55 (7.15, 18.68)
With high SF	1.44 (0.57, 3.63)	1.58 (0.68, 3.63)	15.49 (8.90, 26.97)	18.89 (11.60, 30.78)
**UA ≥ 7.0/6.0 mg/dL (men/women)**				
Without high SF	1	1.48 (1.17, 1.88)	2.77 (2.09, 3.67)	4.54 (3.66, 5.63)
With high SF	1.78 (1.26, 2.52)	2.09 (1.53, 2.87)	4.66 (3.35, 6.49)	6.50 (5.15, 8.21)
**TG ≥ 1.7 mmol/L**				
Without high SF	1	1.91 (1.52, 2.41)	28.12 (21.89, 36.11)	37.71 (30.26, 46.98)
With high SF	2.22 (1.58, 3.14)	3.06 (2.25, 4.17)	50.35 (35.98, 70.46)	72.45 (55.61, 94.38)
**TC ≥ 5.2 mmol/L**				
Without high SF	1	1.43 (1.23, 1.66)	1.63 (1.33, 2.00)	1.88 (1.61, 2.21)
With high SF	1.67 (1.33, 2.10)	2.88 (2.31, 3.58)	2.20 (1.68, 2.89)	3.07 (2.55, 3.68)
**LDL-C ≥ 3.4 mmol/L**				
Without high SF	1	1.65 (1.42, 1.92)	1.41 (1.15, 1.75)	1.53 (1.30, 1.80)
With high SF	1.66 (1.31, 2.10)	2.49 (1.99, 3.11)	1.42 (1.07, 1.89)	1.67 (1.38, 2.02)
**Non-HDL-C ≥ 4.1 mmol/L**				
Without high SF	1	1.86 (1.53, 2.27)	3.17 (2.50, 4.02)	4.24 (3.50, 5.13)
With high SF	2.41 (1.82, 3.19)	3.51 (2.72, 4.52)	4.73 (3.52, 6.35)	7.65 (6.20, 9.43)
**ApoB ≥ 1.17/1.19 g/L (men/women)**				
Without high SF	1	2.17 (1.71, 2.75)	3.21 (2.42, 4.25)	3.96 (3.15, 4.98)
With high SF	1.85 (1.29, 2.64)	3.82 (2.85, 5.13)	5.33 (3.83, 7.40)	5.63 (4.40, 7.20)

a *Adjusted for age, sex, high-sensitivity C-reactive protein and homoeostasis model assessment of insulin resistance*.

*MHNW, metabolically healthy normal weight; MHO, metabolically healthy overweight/obese, MANW, metabolically at-risk normal weight; MAO, metabolically at-risk overweight/obese; SF, serum ferritin; FPG, fasting plasma glucose; UA, uric acid; TG, triglycerides; TC, total cholesterol; LDL-C, low-density lipoprotein cholesterol; Non-HDL-C, non-high-density lipoprotein cholesterol; ApoB, apolipoprotein B100*.

## Discussion

This study has described the characteristics of participants classified according to SF status and body size phenotypes. When compared to subjects without high SF, those with high SF exhibited higher FBG in MHO and MAO groups, higher LDL-C in MHNW and MHO groups, lower HDL-C in the MANW group, and higher UA, TG, TC, non-HDL-C, and apoB in all body size phenotypes. Significant associations were observed for high SF with diabetes in MANW and MAO groups, with unfavorable LDL-C in MHNW and MHO groups, and with other dyslipidemia components (including TG, TC, non-HDL-C, and apoB) and hyperuricemia for all phenotypes. This was the first study to show the proportion of simultaneous presence of the three status, metabolically at-risk, overweight or obese, and high SF, which ranged from 12.84 to 33.39% in individuals with unfavorable CVD risk factors. The clustering of the three status increased with the accumulation of CVD risk factors. For all unfavorable CVD risk factors except LDL-C, the highest ORs were observed in MAO with a high SF group.

Although multiple epidemiological studies have reported correlations between elevated SF levels and CVD risk factors (4, 12, 13, 30, 31), the results are not entirely consistent. Sun et al. found an independent association between elevated SF and diabetes in a prospective study in a Chinese population (31). Similar findings have been reported from prospective cohort studies in Caucasian populations (32–34). The EPIC-Norfolk cohort study established that modest increases in SF could predict incident diabetes independent of confounding factors, especially inflammation indexes (35). The EPIC Postdam study demonstrated an association between SF and diabetic risk independent of biomarkers for hepatic fat accumulation, insulin resistance, and dyslipidemia (36). On the other hand, in the ARIC Study, Jehn et al. found that the significant association between SF and the incidence of diabetes was lost after the data were adjusted for BMI and metabolic syndrome components (37). The majority of studies have established associations between SF and CVD risk factors, as well as the predictive value of SF for risk. Nevertheless, few reports have explained whether or how the relationships have been influenced by the patient's metabolic status or BMI category. This study extended these previous findings by investigating associations based on body size phenotypes and evaluating the separate and combined associations of high SF, overweight/obesity, and those who are metabolically at-risk with CVD risk profiles. Notably, the associations of high SF with the prevalence of diabetes, dyslipidemia, and hyperuricemia varied among individuals within different body size phenotypes. The coincidence of all three status had the strongest associations with unfavorable CVD risk profiles except LDL-C.

Several mechanisms that form the basis for the relationship between SF and diabetes have been demonstrated. IR, as measured using the euglycemic-hyperinsulinemic clamp, has been correlated with body iron stores (10). Other studies have proposed that an iron overload, as reflected by an elevated SF likely induced IR through disturbing inhibition of hepatic glucose production by insulin, and insulin actions in adipose and muscle tissue (38). Furthermore, studies have confirmed the role of hepatic hormones and adipokines in inducing IR. Data from experiments with mice have shown that hepcidin, a key mediator of iron metabolism, could be up regulated by iron and activate the Jak2/STAT3 pathway, which in turn, induced production of suppressor of cytokine signaling-3, which is an inhibitor of insulin signaling (17). Several other studies have demonstrated that induced downregulation of adiponectin, leptin, and upregulation of resistin *via* iron accumulation were causally related to IR (39). Excess iron in specific tissues may play a direct role in diabetes through other mechanisms, including oxidative stress, nitric oxide signaling, and inflammatory cytokines (40). It is worth noting that, in this study, a significant association between SF and diabetes only existed in subjects with a metabolically at-risk status, regardless of the BMI categories. Pancreatic β cells are particularly sensitive to oxygen radicals. Thus, the antioxidant property of SF is the potential reason for increased SF in β cells. The iron overload is not so severe as to induce the rapid apoptosis of β cells as seen with dysmetabolic-based iron overload, but it might have increased glucose levels by promoting β cell failure and reducing insulin synthesis and excretion (41). Although IR and β cell failure contribute to overt diabetes in subjects with iron overload, it is known that metabolic disorders develop from an early stage that precedes β cell deficiency (38). Thus, iron overload might affect the development of diabetes that was tightly linked to a metabolically at-risk status. The present results from the adjusted model were stably independent of hs-CRP and HOMA-IR in metabolically at-risk subjects, which implied the inevitable role of β cell failure in the relationship, although residual confounding was still possible.

Similarly, the association between high SF and hyperuricemia was not entirely explained by IR or chronic low-grade inflammation since it was still significant after the adjustments for HOMA-IR and hs-CRP in all four phenotypes. Except for the induction of elevated UA synthesis by IR (42) and decreased UA excretion (43, 44), xanthine oxidase, a key enzyme in purine metabolism and UA production, has been hypothesized to be responsible for the association between SF and hyperuricemia. Studies have reported that iron overload was followed by elevation and activation of xanthine oxidase *in vivo* and *in vitro*, and tumor necrosis factor-α, interleukin-1, and interleukin-6, which are induced by oxidative stress, take part in the process (45, 46). As a major antioxidant, UA impedes oxidation reactions catalyzed by iron *via* forming a UA-Fe^3+^ complex, and inactivation of antioxidant enzymes such as superoxide dismutase, thus, it is a protective response to iron overload (47). Therefore, it appears that high SF is independently associated with hyperuricemia regardless of the BMI or metabolic status of the subject.

Inconsistencies have been observed for the links between iron overload and dyslipidemia, based on different samples. In a study of adolescents in Korea, positive correlations between SF and TC, LDL-C, and TG only occurred in boys, while a negative association between SF and HDL-C was observed for both genders (48). Other data from young non-obese adults in Korea showed the significant associations of SF with TG and HDL-C only for women (49). In both sexes of a Chinese population, SF was associated with lipid parameters (including TC, LDL-C, TG, and HDL-C) independent of diabetes and IR (12). The previous study using the same cohort confirmed positive correlations of SF with dyslipidemia and unfavorable lipid ratios, especially apoB and non-HDL-C, independent of HOMA-IR (50). Notably, apoB and non-HDL-C are proven independent risk factors for CVD (51). This study extended previously reported results by exploring how such associations performed in different body size phenotypes. It appears that SF was associated with abnormal TG, TC, non-HDL-C, and apoB in all phenotypes. The first proposed mechanism was that iron induced hyperinsulinemia and IR, which in turn contributed to unfavorable TG, LDL-C, TC, and non-HDL-C, and hepatic IR accelerated the synthesis and secretion of apoB through protein-tyrosine phosphatase 1B (52). However, the associations remained significant regardless of the subject's BMI or metabolic status after adjustment for age, sex, hs-CRP, and HOMA-IR. Therefore, alternative underlying mechanisms, except for IR and chronic low-grade inflammation, need to be considered. Studies using *in vivo* rat models suggested reactive oxygen species generated by iron could decrease fatty acid oxidation and enhance lipid transportation *via* suppressed expression of peroxisome proliferator-activated receptor-α and downstream genes (Nrf1, cpt-1α), which promoted hypercholesterolemia and hypertriglyceridemia (53, 54). Also, the up-regulation of microsomal triglyceride transfer protein in iron overloaded rats, which accelerated the processes of apoB production and lipid delivery to the apoB-containing lipoprotein particles, was causally related to the increased levels of apoB, TC, and TG (54, 55). Interestingly, in this study, high SF was associated with elevated LDL-C only in the MHNW and MHO phenotypes, and the associations of high SF with LDL-C and non-HDL-C were more pronounced in the MHNW phenotype compared to subjects with the MANW phenotype. This observation suggested that there were differences in the degree of influence of iron overload and having a metabolically at-risk status on unfavorable LDL-C and non-HDL-C. When a metabolically at-risk status played a more prominent role than iron overload, the iron overload might lose its significance in the relationship with unfavorable LDL-C and non-HDL-C in subjects who were metabolically at-risk. However, few previous studies have examined these underlying mechanisms with respect to this difference. Additional experimental explorations of mechanisms and epidemiological surveys using larger samples should be performed to assess these complex associations.

Although metabolically at-risk participants exhibited higher ORs for unfavorable UA, TG, non-HDL-C, and apoB compared with those with high SF, the presence of high SF was associated with worse CVD risk profiles within the same metabolic status. Clustering of all three status, metabolically at-risk, overweight/obesity, and high SF, had the strongest associations with glycemia, UA, and most lipid profiles. The overlap among the three status increased with the accumulation of CVD risk factors, which might help to identify the high-risk groups earlier. The fact that being metabolically at-risk, overweight/obese, and having high SF have synergistic associations with CVD risk factors suggests that reducing iron overload could be a potential target for the prevention of CVD in subjects with high risk. On the other hand, this study reported considerable dissociation between the three statuses. It is known that elevated BMI is related to the occurrence of insulin resistance and pro-inflammation; BMI can provide a general indication of obesity, but it does not easily distinguish adipose tissue distribution among visceral, ectopic, or subcutaneous accumulation (55). Individuals determined to be metabolically at-risk have been associated with visceral or ectopic adipose tissue distributions and a higher proportion of small adipose cells (56, 57). Therefore, exploring how these three statuses cluster together, and their unique mechanisms of action, could contribute to reducing the adverse effects of each status and scattering them as well.

There are several limitations in this study. First, a cross-sectional study design was not sufficient in drawing any conclusions about causal relationships. Second, a standard 75 g oral glucose tolerance test was difficult to perform in this large-scale population-based survey, Therefore, it is likely that the prevalence of diabetes was underestimated. The use of a calculated HOMA-IR to assess IR was also a limitation.

## Conclusions

The associations of high SF with the prevalence of CVD risk factors, including diabetes, dyslipidemia, and hyperuricemia, vary in individuals with different body size phenotypes. The various synergistic associations of high SF, metabolically at-risk, and overweight/obesity with CVD risk profiles suggest that specific strategies to ensure that the clustered status diverge could be particularly beneficial to prevent CVD.

## Data Availability Statement

The raw data supporting the conclusions of this article will be made available by the authors, without undue reservation.

## Ethics Statement

The studies involving human participants were reviewed and approved by the Institutional Review Board of the University of North Carolina at Chapel Hill, National Institute of Nutrition and Health, China Center for Disease Control and Prevention, and the Human and Clinical Research Ethics Committee of China-Japan Friendship Hospital. The patients/participants provided their written informed consent to participate in this study.

## Author Contributions

GY designed the research. BZ and SL conducted the data analyses. BZ wrote the draft. All authors approved the final manuscript.

## Conflict of Interest

The authors declare that the research was conducted in the absence of any commercial or financial relationships that could be construed as a potential conflict of interest.

## Abbreviations

ApoA1: apolipoprotein A1
ApoB: apolipoprotein B100
BMI: body mass index
BP: blood pressure
CHNS: China Health and Nutrition Survey
CIs: confidence intervals
CVD: cardiovascular disease
FIN: fasting insulin
FPG: fasting plasma glucose
HDL-C: high density lipoprotein cholesterol
HOMA-IR: homeostasis model assessment of insulin resistance
hs-CRP: hypersensitive C-reactive protein
IR: insulin resistance
LDL-C: low density lipoprotein cholesterol
MANW: metabolically at-risk normal-weight
MAO: metabolically at-risk overweight/obesity
Met: metabolic status
MHNW: metabolically healthy normal-weight
MHO: metabolically healthy overweight/obesity
ORs: odds ratios
SF: serum ferritin
TC: total cholesterol
TG: triglycerides
non-HDL-C: non-high-density lipoprotein cholesterol
UA: uric acid.
